# Supplementary material for: Quantification of Tafenoquine and 5,6-Orthoquinone Tafenoquine by UHPLC-MS/MS in Blood, Plasma, and Urine, and Application to a Pharmacokinetic Study
Source: Molecules. 2022 Nov 24;27(23):8186. doi: 10.3390/molecules27238186 (PMC9737280; doi:10.3390/molecules27238186)
Supplement: Supplementary file 1 [file molecules-27-08186-s001.zip › molecules-1970659-supplementary.pdf]

molecules

MDPI

Quantification of Tafenoquine and 5,6-Orthoquinone Tafenoquine by UHPLC-MS/MS in Blood, Plasma, and Urine, and Application to a Pharmacokinetic Study

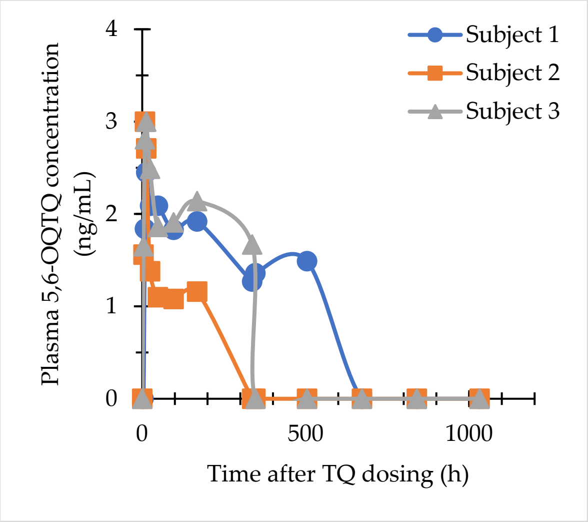

Supplemental Figure S1. 5,6-OQTQ in human plasma from subjects given oral dose of 200 mg TQ base (n=3); Subject 1, (circle line); Subject 2, (square line); Subject 3, (triangle line).

Supplemental Table S1. Cumulative amount excreted (CAE) of TQ and 5,6-OQTQ in human urine from subjects given oral dose of 200 mg TQ base (n=3). * These values were extrapolated below LLOQ and included for illustrative purposes.

	TQ			5,6-OQTQ		
**Hours**	Subject 1	Subject 2	Subject 3	Subject 1	Subject 2	Subject 3
**0**	0	0	0	0	0	0
**24**	4.5*	0.5*	10.8	82.1	41.0	87.9
**48**	60.6	5.1*	89.2	197.1	85.7	247.9
**72**	190.1	8.6*	132.7	395.3	129.4	376.1
**96**	232.1	9.9*	161.1	509.1	160.9	488.4

Supplemental Table S2. Venous blood to plasma TQ concentration ratios at the PK time points for the 3 human subjects.

	**Blood to Plasma Ratio**		
Time (h)	Subject 1	Subject 2	Subject 3
0	N/D	N/D	N/D
4	1.25	1.24	1.21
8	1.04	1.21	1.26
12	1.07	1.12	1.27
24	0.73	1.13	1.25
48	0.93	1.06	1.11
96	0.98	1.05	1.06
168	1.04	1.08	1.13
336	1.03	0.83	1.65
346	1.26	1.42	1.46
504	1.20	1.05	1.02
672	1.17	1.11	1.10
840	1.04	1.24	1.42
